# Comparative Analysis of Legislative Requirements About Patients' Access to Biotechnological Drugs for Rare Diseases in Central and Eastern European Countries

**DOI:** 10.3389/fphar.2018.00795

**Published:** 2018-07-20

**Authors:** Maria Kamusheva, Manoela Manova, Alexandra T. Savova, Guenka I. Petrova, Konstantin Mitov, András Harsányi, Zoltán Kaló, Kristóf Márky, Pawel Kawalec, Bistra Angelovska, Dragana Lakić, Tomas Tesar, Pero Draganic, Mary Geitona, Magdalini Hatzikou, Marian S. Paveliu, Agnes Männik

**Affiliations:** ^1^Department of Organization and Economics of Pharmacy, Faculty of Pharmacy, Medical University-Sofia, Sofia, Bulgaria; ^2^National Council on Pricing and Reimbursement, Sofia, Bulgaria; ^3^Department of Health Policy and Health Economics, Eötvös Loránd University, Budapest, Hungary; ^4^National Institute of Health Insurance Fund Management, Budapest, Hungary; ^5^Syreon Research Institute, Budapest, Hungary; ^6^Faculty of Health Sciences, Institute of Public Health, Jagiellonian University Medical College Kraków, Kraków, Poland; ^7^Department of Pharmacy, Faculty of Medical Sciences, University Goce Delcev- Stip, Štip, Macedonia; ^8^Department for Social Pharmacy and Pharmaceutical Legislation, Faculty of Pharmacy, University of Belgrade, Belgrade, Serbia; ^9^Department of Organization and Management in Pharmacy, Faculty of Pharmacy, Comenius University, Bratislava, Slovakia; ^10^Croatian Agency for Medicinal Products and Medical Devices, Zagreb, Croatia; ^11^Department of Social and Educational Policy, School of Social Sciences, University of Peloponnese Tripoli, Tripoli, Greece; ^12^Department of Pharmacology and Pharmaeconomics, Faculty of General Medicine, Titu Maiorescu University, Bucharest, Romania; ^13^Institute of Family Medicine and Public Health, University of Tartu, Tallinn, Estonia

**Keywords:** CEE countries, orphan medicinal products, reimbursement, rare diseases, biotechnology

## Abstract

**Objectives:** The aim of the study was to compare the access of patients with rare diseases (RDs) to biotechnological drugs in several Central and Eastern European countries (CEECs). We focused on the legislative pricing and reimbursement requirements, availability of biotechnological orphan medicinal products (BOMPs) for RDs, and reimbursement expenditures.

**Methods:** A questionnaire-based survey was conducted among experts from 10 CEECs: Bulgaria, Croatia, Estonia, Greece, Hungary, Poland, Romania, Slovakia, Serbia, and Macedonia. The legal requirements for reimbursement and pricing of BOMPs were collected. All BOMPs and medicines without prior orphan designations were extracted from the European list of orphan medicinal products, 2017. The reimbursement status of these medicinal products in 2017 in the public coverage of the included CEECs as well as the share of their costs in relation to the total public pharmaceutical spending for the period from 2014 to 2016 were defined.

**Results:** Our survey revealed that some differences in the legal requirements for pricing and reimbursement of BOMPs amongst the countries included in the study. All European Union countries have developed and implemented pharmacoeconomic guidelines with or without some specific reimbursement requirements for orphan medicinal products. Cost-effectiveness analysis, cost-utility analysis, Markov models, meta-analysis, and discount levels of costs and results were required only in Bulgaria, Poland and Hungary. The number of reimbursed BOMPs and biotechnological medicinal products for RDs without prior orphan designation was the highest in Hungary (17 and 40, respectively). Patient-based reimbursement schemes were available only in Hungary for 11 out of 17 BOMPs. Poland and Greece have the highest pharmaceutical expenditure of reimbursed BOMPs with are ~214 million and 180 million EUR, respectively in the observed period from 2014 to 2016. High proportion of the pharmaceutical expenditure on the reimbursed biotechnological medicinal products for RDs for the observed period 2014–2016 is presented in Bulgaria and Slovakia.

**Conclusions:** The non-European Union CEECs face a significant delay in the legal implementation of pharmacoeconomic guideline for assessment of BOMPs. The access to BOMPs is similar among the observed CEECs and the countries with the best access are Hungary and Greece. The influence of BOMP expenditures on the budget in the individual countries is significant.

## Introduction

The definitions for rare diseases vary across the regions, but the major classification criterion is associated with the number of affected patients: not more than 5 in 10,000 in the European Union and in Canada and fewer than 200,000 people in the USA (Stoimenova et al., 2011; Chicevaliev and Aleksovska, 2016; Lacoste, 2018). Despite the limited number of individuals affected by a particular rare disease the total number of patients represents a significant percent (Schieppati et al., 2008). More than 80% of all classified as rare diseases (RDs) are of genetic origin, which defines the main purpose of the scientists: to carry on more and more genetic research in order to discover the intimate RDs pathogenic mechanisms (Das et al., 2008; Altshuler et al., 2010; de Vrueh et al., 2013). The lack of available effective and safe treatment for many rare diseases is considered as an enormous problem (Elliott and Zurynski, 2015)^1^^,^^2^, because every citizen should be provided with quality medical care. On the other side the cost-effectiveness of orphan medicines is difficult to be established. A lot of efforts are being made so as innovative and targeted medicines for the individual patients to be developed and authorized in short terms, but this still does not solve the affordability issues (Vella Bonanno et al., 2017). The number of medicines, produced through biotechnological methods, such as enzymes, cytokines, hormones, monoclonal antibodies, etc. has been increasing tremendously in the recent years ensuring effective and safe treatment targeting specific mediators (Bruggemeier, 2006; Bellomo et al., 2017; Geynisman et al., 2017). In the recent years, gene transfer technology has been developed and implemented for the therapy of rare genetic disorders (Han et al., 2017; Mavilio, 2017). Developing cell, gene, nanobiotechnology-based and other biotechnology therapies ensure meeting the unmet medical need in a number of rare diseases (Brooks et al., 2016; Nance et al., 2018).

Despite the significant clinical benefits that the biologic treatments offer their costs are much higher than in case of the small molecules. Therefore, they have a significant financial impact on the total health care budget as it is expected to reach $75–90 billion in spending by 2021^3^. This could put additional pressure on the healthcare budgets which are limited especially in the low and middle-income countries and therefore could limit the patients' access to therapy (Moorkens et al., 2017). Kawalec et al. revealed that the total expenditures on the reimbursement of biologic drugs in the Central and Eastern European Countries (CEECs) were seriously increasing for the period 2014–2015 as well as more than 80% of the value in 2014 and more than 70% in 2015 was covered by the reimbursement of original drugs in the same countries (Kawalec et al., 2017). The mechanisms for cost reduction such as authorization of biosimilars are implemented in order to be provided an adequate patients' access to treatment (Petigara and Anderson, 2008). For example, the growth rate in the expenditures for biotechnological drugs could be decreased by ~5% through implementation of treatment with biosimilars. Restrictive requirements about spending control mechanisms are available in Europe such as the need for strict drug cost-value assessment, defining of budget caps and pharma payback schemes, price negotiations etc.

Health technology assessment and specific pharmacoeconomic criteria are applied in every country so as the cost-effectiveness of biologics for rare diseases have to be assessed for the purposes of their inclusion in the drug list covered by public funds. Moreover, a higher threshold than the conventional one is recommended for this group of products in some countries (Longson, 2017; Kamusheva et al., 2018).

Besides, the launching of biologics is accompanied by a number of challenges and one of them is associated with the provision of an equitable access facing differences in pricing and reimbursement policies in every country (Ling et al., 2014; Lybecker, 2016). Therefore, our main aim was to compare an access to biotechnological drugs for patients with rare diseases (RDs) in several CEECs. We have focused on the legislative pricing and reimbursement requirements, the availability of biotechnological orphan medicinal products (BOMPs) for RDs and on their reimbursement expenditures. We explored several research questions as the availability of biotechnological orphan medicines in the reimbursement system in the considered countries, their coverage from public resources, and market access in reference to Health Technology Assessment (HTA) requirements.

## Materials and methods

In the current comparative analysis, we included biotechnological OMPs, which were extracted from the latest version of the List of orphan drugs published by Orphanet in July 2017^4^. Both lists of OMPs with and without prior orphan designation were taken into consideration and the analysis was presented separately. The comparative questionnaire-based analysis was made for 24 biotechnological orphan medicines and for 49 biotechnological medicines without prior orphan designation.

A questionnaire-based survey was conducted among leading experts from twelve CEEC: Bulgaria, Croatia, Estonia, Greece, Hungary, Poland, Romania, Slovakia, Serbia, Macedonia, the Czech Republic, and Latvia Only ten out of these 12 countries gave their consent to take part in the survey and sent the questionnaire with respective answers. Serbia and Macedonia as non-EU member states were selected so as a comparison between non-EU and EU countries to be performed. The communication was realized by e-mail using English language and the subsequent answers had been received for a period of several months: from October 2017 to February 2018.

The questionnaire written in English was standardized according to the aim of the project and examined several aspects:

1. What are the existing legal requirements for pricing and reimbursement of biotechnological orphan medicinal products in the selected CEEC?
  - Is there a specific part in the pharmacoeconomic guideline about biotechnological orphan medicinal products (BOMPs)?
  - What are the basic principles of the included in the survey CEEC's pricing system?
  - Are there any discounts for the purposes of BOMPs expenditures control?
  - Which are the specific requirements for reimbursement of BOMPs?
2. What are the requirements for the preparation of pharmacoeconomic/HTA dossiers of BOMPs?
  - Is there a separated threshold for cost-effectiveness assessment of BOMPs?
  - Which are the main pharmacoeconomic methods, which are obligatory for BOMPs?
  - Which are the additional non-economic considerations taken into account for BOMPs?
3. Which are the BOMPs included in every local positive drug list?
4. Which are the biotechnological medicinal products without prior orphan designation intended to treat rare diseases which are included in every local positive drug list?
5. What are the pharmaceutical expenditures paid by public funds in the selected CEEC and what the share of BOMPs from the total expenditures is for a 3-year period of time 2014–2016?

Lastly, the statistical analysis using MedCalc software was performed on the basis of total amount of data on the reimbursed BOMP collected in the survey. The share of BOMPs expenditures in relation to total pharmaceutical expenditures for the observed period of time was calculated for each individual country as well as the number of BOMPs, reimbursed in all considered CEEC. The collected information from each country was extracted, classified and analyzed by the experts.

## Results

### Pricing and reimbursement requirements for BOMPs in selected CEE countries

The essential legal requirements for reimbursement of biotechnological orphan medicinal products in the selected CEECs were summarized and presented in Table 1. All EU member states included in the analysis, except Greece and Romania, have implemented pharmacoeconomic and Health Technology Assessment guidelines in accordance with the recommendations. The Hungarian Guidelines were first published in 2003 (Szende et al., 2002) and the last revision was issued in February 2017 (Gyógyszereink, 2017). Balkan countries outside the EU, Serbia, and Macedonia, have not followed any official guidelines yet.

**Table 1:** Legal requirement about reimbursement and pricing of orphan medicinal products with a focus on the biotechnological drugs.

	**Availability of pharmacoeconomic/HTA guidelines**	**Special section for rare diseases and/ or orphan drugs in the guideline**	**Specific requirements for reimbursement decision**	**Specific requirements for reimbursement**	**Deadlines for making reimbursement decision**	**Pricing system**	**Discounts**
Bulgaria	+/+	+/+	The same as for all medicines	Clinical indication (e.g., state of the disease); Limitation in the number of patients; Age of the patients.	90 days for HTA appraisal; Plus 90 days for pricing and reimbursement decision.	External reference pricing, lower from 17 EU reference countries	Obligatory no less than 10% and confidential
Croatia	+/+	+/+	The same as for all medicines	According to the guidelines	180 days for HTA appraisal and for pricing and reimbursement decision	Reference pricing, a combination of 5 EU reference countries	Compulsory no < 10% and also confidential
Estonia	+/+	–/–	The same as for all medicines	Clinical indications need to be listed in a specific governmental act.	180 days	Price cannot be higher than in Latvia, Lithuania and Slovakia.	Confidential additional discounts are possible.
Greece	-/-	–/–	–/–	Limited to exact approved indication	4 months for pricing 2–3 months for reimbursement.	Reference pricing: the average of the three lowest between 27 EU countries	Obligatory 14–30% scaled rebate depending on volume
Hungary	+/+^**^	–/–	The same as for all medicines	Clinical indication (e.g., state of the disease); Limitation in the number of patients; Age of the patients.	90 days for Pricing & Reimbursement; 43days for the HTA appraisal	External Referencing pricing	Confidential
Macedonia	-/-	–/–	The same as for all medicines	Non-defined	Non-defined	Public procurement by the Ministry of Health-criterion-the lowest price	-
Poland	+/+	–/–	The same as for all medicines	No specific requirements	90 days for reimbursement decision; 180 days for pricing and reimbursement decision according to the Transparency Directive	External referencing pricing: 31 UE and EOG reference countries	No formal discounts; More popular and more often used are Risk Sharing Schemes (RSSs)
Romania	–/–	–/–				–/–	–/–
Serbia	–/–^*^	–/–	The same as for all medicines	Special agreement (PAS)—volume-, value-cap, risk-, or cost-sharing. Limitation in the number of patients, indication, the severity of disease, etc.	For pricing—not specially defined, but not more than 90 days; For reimbursement-−120 days.	Reference pricing: *First* basket: 3 EU countries; *Second* basket: EU country manufacturing drug or EU countries that have drug on the market.	Depends on negotiated price and terms; Confidential.
Slovakia Ministry of Health (2011a). [Act No. 363/2011]	+/-	+/+	The same as for all medicines	Prescription restrictions; Medical indication restrictions; Restricted prescription only in specialized hospitals	180 days for pricing and reimbursement decision according to the Transparency Directive	External reference pricing methodology. The *average of the three lowest prices in the EU countries*	Confidential additional discounts are possible.

* *Not complete guideline, more certain aspects covered in legal Act*.

** *The guideline is also available In English: http://www.ogyei.gov.hu/dynamic/Gyogyszereink_2017_1_eng.pdf. Ref: Professional healthcare guideline on the methodology of health technology assessment. (Gyógyszereink, 2017). 67. 1. (special issue). 1-23*.

**Table 2:** Requirements about the pharmacoeconomic analysis/HTA document performed for the purposes of reimbursement of OMPs.

**Country**	**Pharmacoeconomic method applied (CEA, CUA etc.)**	**Separated ICER threshold for orphan drugs**	**Is budget impact analysis requested in the guideline?**	**Are the expenditures for comparative treatments taken into account?**	**Are Markov models obligatory? (if eligible)**	**Meta-analysis of conducted clinical trials is requested (if eligible)**	**What is the discounted level for costs?**	**What is the discounted level for clinical results?**	**Are the HTA appraisals from other countries valuable for the final decision?**	**Assessment of social and economic burden of the disease, its rarity and seriousness**
Bulgaria	CEA, CUA	Conventional threshold (1–3 times GDP/capita)	+	+	+	+	5%	5%	+	+
Croatia	BIA, CEA, CUA	Conventional threshold (1–3 times GDP/capita)	+	+	+	+	5%	5%	+	+
Estonia	CMA, CEA, CUA	No threshold	+	+	-	+	5%	5%	+	+
Greece	CEA	Not yet defined for any medication	+	–	Not yet defined	It is allowed but not explicitly defined.	Not yet defined	Not yet defined	+	+/–
Hungary	CEA, CUA	The same threshold as for non-orphan drugs (3 times GDP/capita)	+	+	+	+	3,7%	3,7%	+	+
Macedonia	–	–	–	–	–	–	–	–	–	–
Poland	CEA CUA	The same threshold as in case of non-orphan drugs 3 × GDP/capita	+	+	+	+	5%	3.5%	+/-	+
Romania	-	NA	+	+	–	–	NA	NA	+	–
Serbia	CMA; CEA; CUA	-	+	+	Not defined	Not defined	Not defined	Not defined	+/-	+/-
Slovakia Ministry of Health (2011b). [Decree No. 422/2011]	CMA, CEA, CUA	The conventional thresholds are not applied for orphan drugs (number of patients lower than 1: 50 000	+	+	–	–	5%	5%	+	+

A special section of the guidelines, indicated exclusively for orphan medicines is available in just a few countries: Bulgaria, Slovakia and Croatia. Moral and ethical considerations (equality, fair and timely adequate access to safe and effective treatment), some limitations about clinical indication (e.g., state of the disease, predefined biochemical parameters, lack of alternative treatment), as well as the number of patients or the age of the patients, providing Markov model for presenting the outcomes etc. are mentioned in the additional section about OMPs of the Bulgarian guidelines (Ministry of Health, Bulgaria, Regulation No. 9 of 01.12. 2015 on the conditions and procedures for conducting health technology assessment). The conventional thresholds set in Slovakia (lower threshold (λ1): 35 times average monthly salary and the upper threshold (λ2): 41 times average monthly salary) are not applicable for orphan drugs indicated in the therapy of rare diseases, where number of patients eligible for treatment with a medicinal product based on the indication approved in marketing authorization is lower than 1: 50,000 in the Slovak republic. In such cases, there is no need for pharmaco-economic analysis.

Reimbursement decisions are based on the conventional requirements which are valid for all medicines. The conventional pharmacoeconomic analysis and HTA dossiers include evidence for cost-effectiveness and present the budget impact of the new medicines taking into account the current treatment strategies. The reimbursement of BOMPs in some countries such as Bulgaria, Serbia, Hungary, Estonia and Greece is based on specific additional criteria such as clinical indications, the age of patients, limitation in the number of treated patients and severity of the disease. These adopted limitations ensure an adequate cost containment measure for the local pharmaceutical budgets.

The deadlines for pricing and reimbursement (P&R) decisions for the EU members are according to the Transparency Directive −180 days. In Serbia it is 120 days for reimbursement and 90 days for pricing whereas no timeline is specified in Macedonia. In Hungary the duration of the P&R procedure itself is 90 days. Moreover, 43 days are needed for the HTA Office within the whole P&R procedure to evaluate the dossier and make a suggestion for reimbursement. In Bulgaria for all new INNs the procedure is extended to 180 days and included an opinion given by the HTA Committee and final decision made by the National Council on Pricing and Reimbursement.

External reference pricing is valid for all medicinal products as well as for BOMPs in all considered countries. However, the number of chosen reference countries and the external pricing rules differ among the countries. In Bulgaria the manufacturing price must be not higher than the lowest price among the 17 reference countries. Whereas MPs price in Greece is defined as the average of the three lowest in 27 European Union countries. Croatia uses only 5 EU countries as reference countries. The final price in Slovakia may not exceed the average of the three lowest prices of the same medicine available on pharmaceutical markets across the European Union (EU). In Hungary, Norway and Switzerland there are also considered as reference countries as the lowest price within the basket of countries is defined as the reference list price. Poland uses 31 countries of the European Union and European Economic Area as reference countries and the price in Poland should be lower than the lowest price of the drug in the reference countries. Estonia compares the manufacturer price only with 3 countries as it should be lower than the same price in Latvia, Lithuania, and Slovakia. The criterion used in Macedonia is the lowest price as the price is negotiated and provided through public procurement by the Ministry of Health. The other non-EU country included in the survey, Serbia, has two baskets: the first basket consists of 3 EU countries, while the second one includes European country where the product is manufactured or EU countries that have the same MP on the market.

Obligatory confidential discounts on different levels are applied in most countries. Besides, varieties of Risk Sharing Schemes are adopted in Greece and Poland valid for majority medicinal products.

### Structure of the pharmacoeconomic analysis/HTA dossier in the considered CEEC

The main pharmacoeconomic methods used in almost all CEEC countries (except for Romania) are cost-effectiveness, cost-utility analysis and in some cases—cost-minimization. A proxy scorecard is applied in Romania, so CEA and CUA are not applicable and the reimbursement decision is mainly based on that taken in other countries such as Germany, UK, France, and Scotland.

Differences in the thresholds for both orphan medicines and non-orphan medicines are not defined in almost all CEEC except Slovakia. Estonia, Greece, Romania, and Serbia do not use any threshold for cost-effectiveness assessment of all MPs. In Bulgaria, Poland, Hungary, and Croatia the threshold is the same as for all non-orphan medicines: 1–3 × gross domestic product per capita (GDP/ capita).

Budget impact analysis and clinical and cost comparisons with appropriate reference drugs are valid for all considered countries. Application of Markov models is obligatory in some countries (Bulgaria, Croatia, Hungary, Poland) while optional in others (Slovakia, Estonia). Obligatory modeling is not yet implemented in the guidelines in Greece, Serbia and Romania. Provision of meta-analysis (where eligible) is also a requirement, defined in the Bulgarian, Croatian, Hungarian, Polish and Estonian pharmacoeconomic and HTA guidelines. The discounted level is strictly defined to be 5% for both costs and clinical results in most of the countries. The only exception is Poland, where the level for clinical results is lower than the level for the costs−3.5 vs. 5%.

The final reimbursement decision is a result of a detailed and transparent appraisal, which in some CEEC is based on reports published in other countries—UK, Germany, France, and Scotland. If NICE, HAS or IQWIG gave a negative recommendation the procedure in Bulgaria is terminated with a negative reimbursement decision. On the other side, these reports are just additional aspects considered in Poland as they did not directly influence the final reimbursement recommendations or reimbursement decisions.

The social and economic burden of the disease, its rarity and seriousness are considered in all countries. They should be represented in the dossier and the final decision is based on a set of economic, social and ethical considerations.

### Reimbursed BOMPs and biotechnological MPs without orphan designation in CEEC

**Figure 1:**
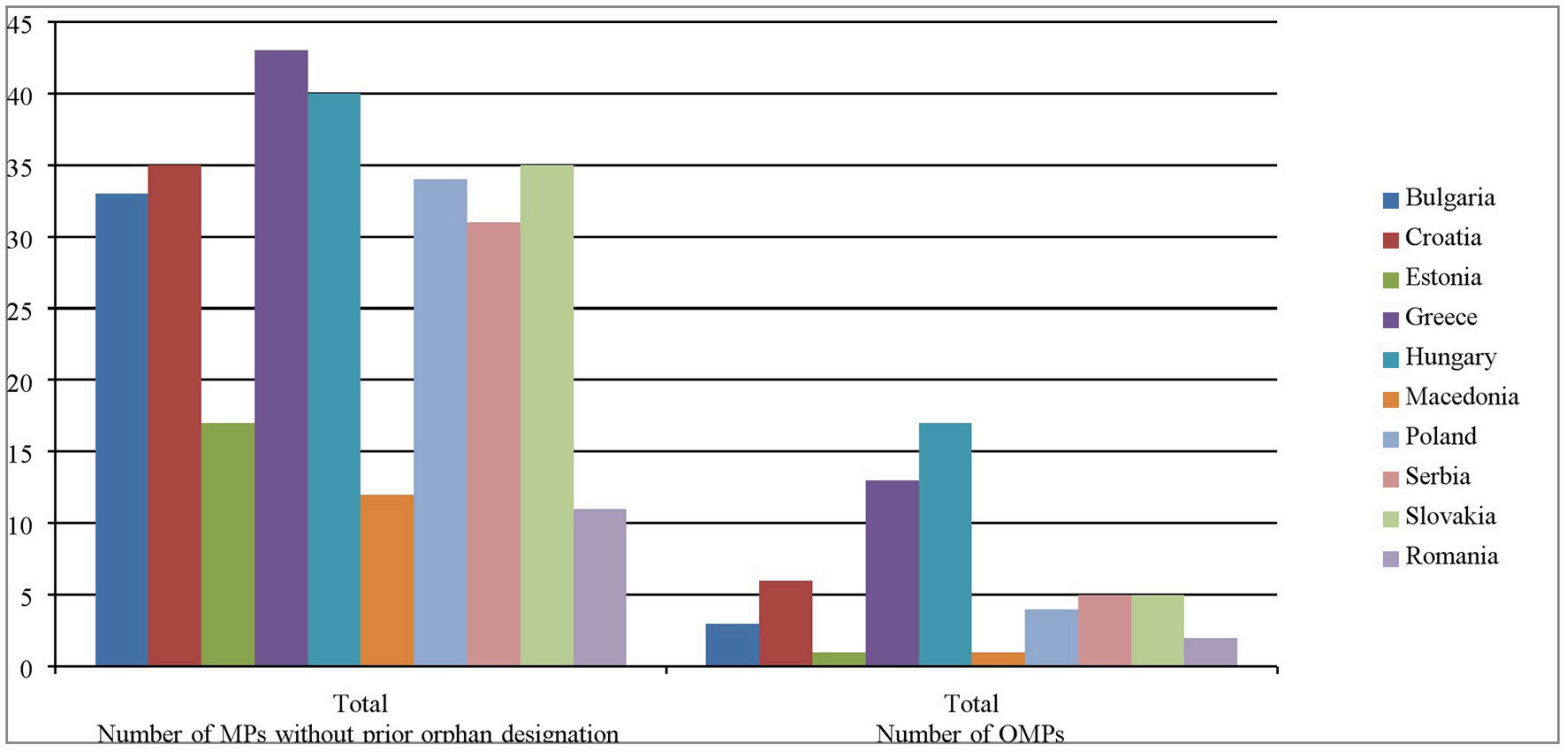
Number of biotechnological orphan medicines and biotechnological MPs for rare disease without prior orphan designation in the selected CEEC. MPs, medicinal products; OMPs, orphan medicinal products.

According to the List of Orphan Drugs in Europe, published in July, 2017 the number of biotechnological drugs is 24 and the number of biotechnological MPs without prior orphan designation is 49. As a result of comparison between this list and the reimbursement lists available in all included in the current analysis CEEC, Hungary and Greece are the countries with the highest number of biotechnological orphan (71 and 54%, respectively) and non-orphan (82 and 88%, respectively) medicinal products for rare diseases (Tables 3, 4, Figure 1). The countries with the most limited access to biotechnological orphan medicines are Macedonia and Estonia with only one medicine reimbursed, followed by Romania, Serbia (*n* = 2), Bulgaria (*n* = 3), Slovakia (*n* = 4), and Croatia (*n* = 7). Using the first level of Anatomical Therapeutic Chemical classification system (ATC), the prevailing biotechnological OMPs are from group ‘L – Antineoplastic and immunomodulating agents’ (9 medicinal products). In Hungary some medicines are not included in the positive list but they are available and reimbursed through the so-called ‘Patient-based reimbursement system’ (*n* = 11).

**Table 3:** Biotechnological orphan medicinal products intended to treat rare diseases included in the local positive drug lists.

**INN**	**ATC code**	**Bulgaria**	**Croatia**	**Estonia**	**Greece**	**Hungary**	**Macedonia**	**Poland**	**Romania**	**Serbia**	**Slovakia**
Cerliponase alfa	A16AB17	–	–	–	–	–	–	–	–	–	–
sebelipase alfa	A16AB14	–	–	–	–	–	–	–	–	–	–
teduglutide	A16AX08	–	–	–	–	–	–	–	–	–	–
asfotase alfa	A16AB13	–	–	–	–	PBR^***^	–	–	–	–	–
elosulfase alfa	A16AB12	–	–	–	–	PBR^***^	–	–	–	Registered, not reimbursed	-
velaglucerase alfa	A16AB10	–	+	-	+/HC^**^	PBR^***^	–	+	–	–	+
Eftrenonacog alfa	B02BD04	–	–	–	+/HC^**^	+	–	–	–	–	–
Human coagulation factor X	B02BD13	–	–	–	-	+	+	–	–	–	Only in the combination: ATC Group B02BD01
Defibrotide	B01AX01	–	–	–	+/HC^**^	–	–	–	–	–	–
albutrepenonacog alfa	B02BD04	–	–	–	+/HC^**^	+	-	-	-	-	-
romiplostim	B02BX04	+	+		+/AC^*^	+	-	-	+	+/HC^**^	+
Alipogene tiparvovec	C10AX10	–	–	–	–	–	–	–	–	–	–
afamelanotide	D02BB02	–	–	–	–	PBR^***^	–	–	–	–	–
mecasermin	H01AC03	–	–	–	+/AC^*^	–	–	+	–	–	–
parathyroid hormone	H05AA03	–	–	–	-	–	–	–	–	–	–
Brentuximab vedotin	L01XC12	+	+	–	+/HC^**^	+	–	+	–	+/HC^**^	–
Ofatumumab	L01XC10	+	+	+/AC^*^	+/HC^**^	PBR^***^	-	–	+	Registered, not reimbursed	+
Blinatumomab	L01XC19	–	–	–	+/HC^**^	PBR^***^	-	–	–	–	–
daratumumab	L01XC24	–	–	–	+/HC^**^	PBR^***^	-	–	–	–	–
Dinutuximab beta	L01XC16	–	–	–	-	PBR^***^	-	–	–	–	–
Obinutuzumab	L01XC15	–	+	–	+/HC^**^	+	-	+	-	Registered, not reimbursed	+
olaratumab	L01XC27	–	-	–	–	PBR^***^	-	–	–	–	–
eculizumab	L04AA25	–	+	–	+/HC^**^	PBR^***^	-	–	–	–	–
siltuximab	L04AC11	–	-	–	+/HC^**^	PBR^***^	-	–	–	–	–
Number of BOMPs		3	6	1	13	17	1	4	2	5	4

*ATC, Anatomical Therapeutic Chemical Classification System*.

* *AC, ambulatory care*.

** *HC, hospital care*.

*** *PBR: Not in the positive list, but available and reimbursed through ‘Patientbased reimbursement system*.

**Table 4:** Biotechnological medicinal products without prior orphan designation intended to treat rare diseases included in the local positive drug lists.

	**ATC code**	**Bulgaria**	**Croatia**	**Estonia**	**Greece**	**Hungary**	**Macedonia**	**Poland**	**Romania**	**Serbia**	**Slovakia**
laronidase	A16AB05	Only price; it's not reimbursed	+	–	+/HC^**^	PBR^***^	–	+	+	Registered, not reimbursed	+
imiglucerase	A16AB02	+	+	–	+/HC^**^	PBR^***^	–	+	–	Registered, not reimbursed	+
idursulfase	A16AB09	+	+	–	+/HC^**^	PBR^***^	–	+	+	Registered, not reimbursed	-
agalsidase beta	A16AB04	+	+	+/HC	+/HC^**^	+	–	–	+	Registered, not reimbursed	+
Alglucosidase alfa	A16AB07	+	+		+/HC^**^	PBR^***^	–	+	+	Registered, not reimbursed	+
Galsulfase	A16AB08	Only price; it's not reimbursed	+	-	+/HC^**^	PBR^***^	–	+	–	–	–
agalsidase alfa	A16AB03	+	+	+/HC^**^	+/HC^**^	PBR^***^	–		+	-	+
octocog alpha	B02BD02	-	+	-	+/HC^**^	+	-	+	–	+	+
nonacog alpha	B02BD04	+			+/HC^**^	+		+		+	
Human protein c	B01AD12	–	–	–	+/HC^**^	-	–	–	–	–	Only in the combination:ATC Group B02BD01
C1 inhibitor (human)	B02AB03	+	–	–	+/HC^**^	+	+	+	–	–	+
efmoroctocog alfa	B02BD02	+	–	–	+/HC^**^	+	–	+	–	–	+
human coagulation factor IX	B02BD13	+	+	+	+/HC^**^	+	+	+		+	+
turoctocog alpha	B02BD02	+	+	-	+/HC^**^	+	–	+	–	Registered, not reimbursed	+
eptacog alpha	B02BD08	+	+	–	+/HC^**^	+	+	-	-	+	+
catridecacog	B02BD11	–	–	–	+/HC^**^	+	–	–	–	–	–
Susoctocog alfa	B02BD14	–	–	–	–	–	–	–	-	–	–
Moroctocog alpha	B02BD02	+	+		+/HC^**^	+				+	+
human alpha1- proteinase inhibitor	B02AB02	-	+	–	+/HC^**^	–	–	–	–	–	–
Nonacog gamma	B02BD04	-	-	–	–	–	–	+	–	–	–
conestat alfa	B06AC04	+	+			+	-	+	–	–	+
human coagulation factor viii/ von willebrand factor	B02BD06	+	+	+	+/HC^**^	+		+		+	+
Evolocumab	C10AX13	+	–	–	+/AC^*^	–	–	–	–	Registered, not reimbursed	+
follitropin alfa	G03GA05	+	+	+/AC^*^	+/AC^*^	+	+	+	-	+	+
follitropin beta	G03GA06	+	+	+/AC^*^	+/HC^**^	+	+	+	-	+	+
somatropin	H01AC01	+	+	+/AC^*^	+/HC^**^	+	+	+	-	+	+
Pegvisomant	H01AX01	+	+	–	+/HC^**^	PBR^***^	–	–	+	–	+
thyrotropin alfa	H01AB01	+	+	–	+/HC^**^	+	–	+	+	Registered, not reimbursed	+
human normal immunoglobulin	J06BA	+	+	+/HC^**^	+/HC^**^	+	+	+	+	+	+
human hepatitis b immunoglobulin	J06BB04	+	+	+/HC^**^	+/AC^*^	+	+	-	-	+	-
adalimumab	L04AB04	+	+	+/HC^**^	+/AC^*^	+	-	+	–	+	+
bevacizumab	L01XC07	+	+	+/HC^**^	+/AC^*^	+	-	+	–	+	+
elotuzumab	L01XC23	-	-	-	-	-	-	-	–	–	–
etanercept	L04AB01	+	+	+/HC^**^	+/HC^**^	+	-	+	–	+	+
cetuximab	L01XC06	+	+	+/HC^**^	+/HC^**^	+	-	+	–	+	+
Filgrastim	L03AA02	+	+	+/HC^**^	+/AC^*^	+		+	–	+	+
Trastuzumab	L01XC03	+	+	+/HC^**^	+/HC^**^	+	+	+	–	+	+
Canakinumab	L04AC08	-	-	-	+/AC^*^	+	-	-	–	-	-
interferon alpha-2b	L03A B05	-	+	-	+/AC^*^	+	+	+	+	+	+
Pembrolizumab	L01XC18	-	-	-	+/HC^**^	+	-	+	-	+	-
anakinra	L04AC03	-	+	-	+/HC^**^	-	-	+	-	-	-
Rituximab	L01XC02	+	+	+/HC^**^	+/HC^**^	+	+	+	+	+	+
pegaspargase	L01XX24	–	+	-	-	-	-	+	-	-	-
nivolumab	L01XC17	–	-	+/HC^**^	+/HC^**^	+	-	+	-	Registered, not reimbursed	-
abatacept	L04AA24	–	–	–	+/HC^**^	+	–	–	-	–	+
Tocilizumab	L04AC07	+	+	–	+/HC^**^	+	–	+	–	+	+
golimumab	L04AB06	+	+	–	+/AC^*^	+		+	–	+	+
Asparaginase	L01XX02	–	+	–	+/HC^**^		+	+	–	–	+
ibritumomab tiuxetan	V10XX02	–	–	–	–	+	–	–	–	–	+
Number of BOMPs		**33**	**35**	**17**	**43**	**40**	**12**	**34**	**11**	**31**	**35**

ATC, Anatomical Therapeutic Chemical Classification System.

* AC, ambulatory care.

** HC,hospital care.

*** *PBR: Not in the positive list, but available and reimbursed through ‘Patient-based reimbursement system*.

The share of antineoplastic and immunomodulating drugs among all biotechnological medicines for RDs without prior orphan designation is the highest—it is around 39%. Most reimbursed medicines in almost all observed CEEC are from the group of antineoplastic agents: 29% in Bulgaria, 37% in Croatia, 37% in Greece, 44% in Poland. Greek healthcare fund reimbursed 43 biotechnological MPs without prior orphan designation, most of which are in the group of antineoplastic (37%), followed by blood products−28%. Surprisingly, the number of reimbursed medicines in Romania is lower than in Serbia, which is non-European union (non-EU) member state−11 vs. 22 MPs. Poland, Bulgaria, Croatia, and Slovakia have included almost equal number of biotechnological non-orphan drugs−34, 31, 35, and 35.

Some of the biotechnological drugs are not reimbursed but are authorized and available on the market with a price. Therefore, patients have restricted affordability to these medicinal products due to their high price and lack of reimbursement status.

### Reimbursement expenditures for BOMPs in CEE countries

**Figure 2:**
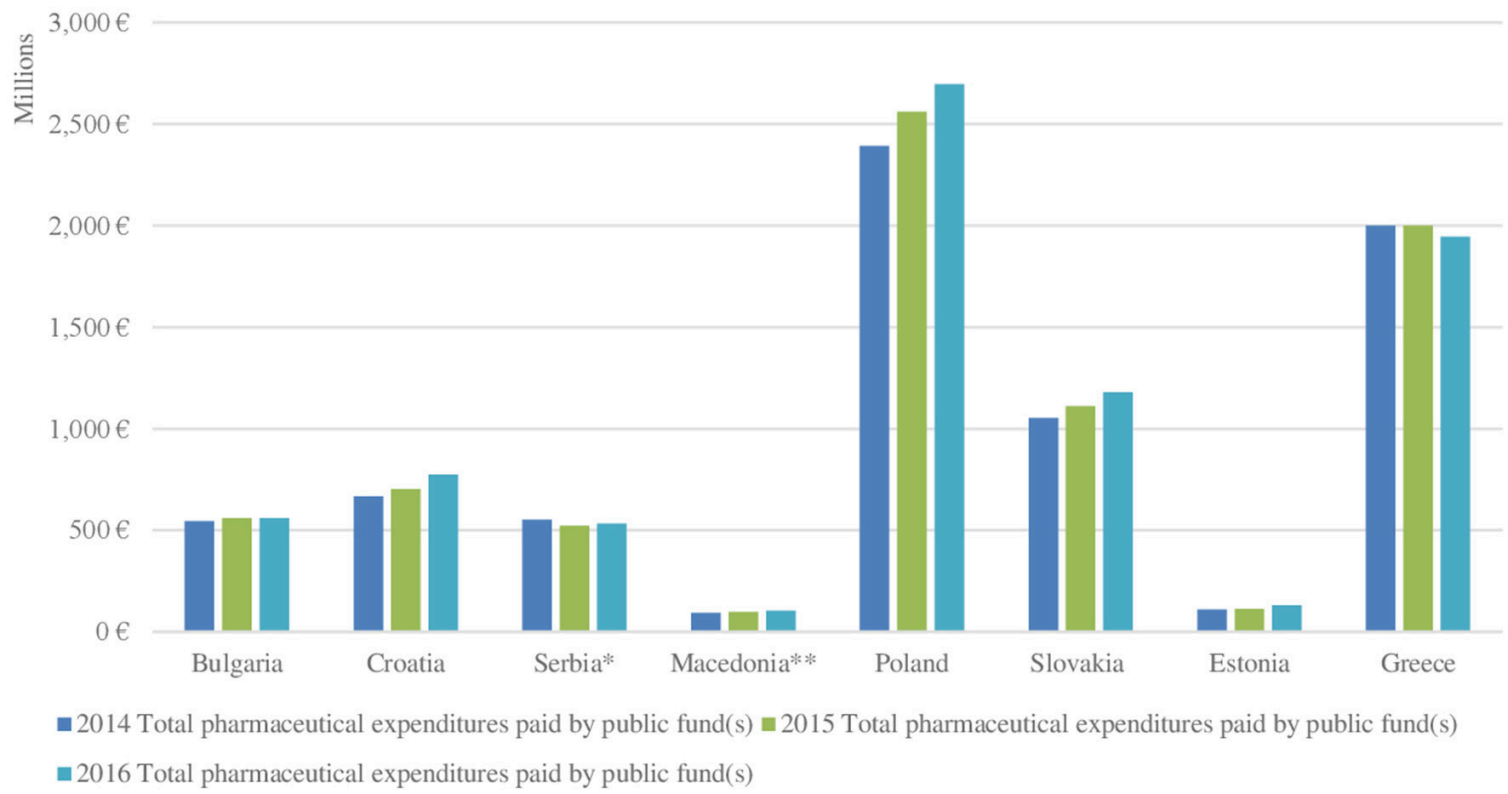
Total pharmaceutical expenditures paid by the public fund in each country in 2014–2016.

**Figure 3:**
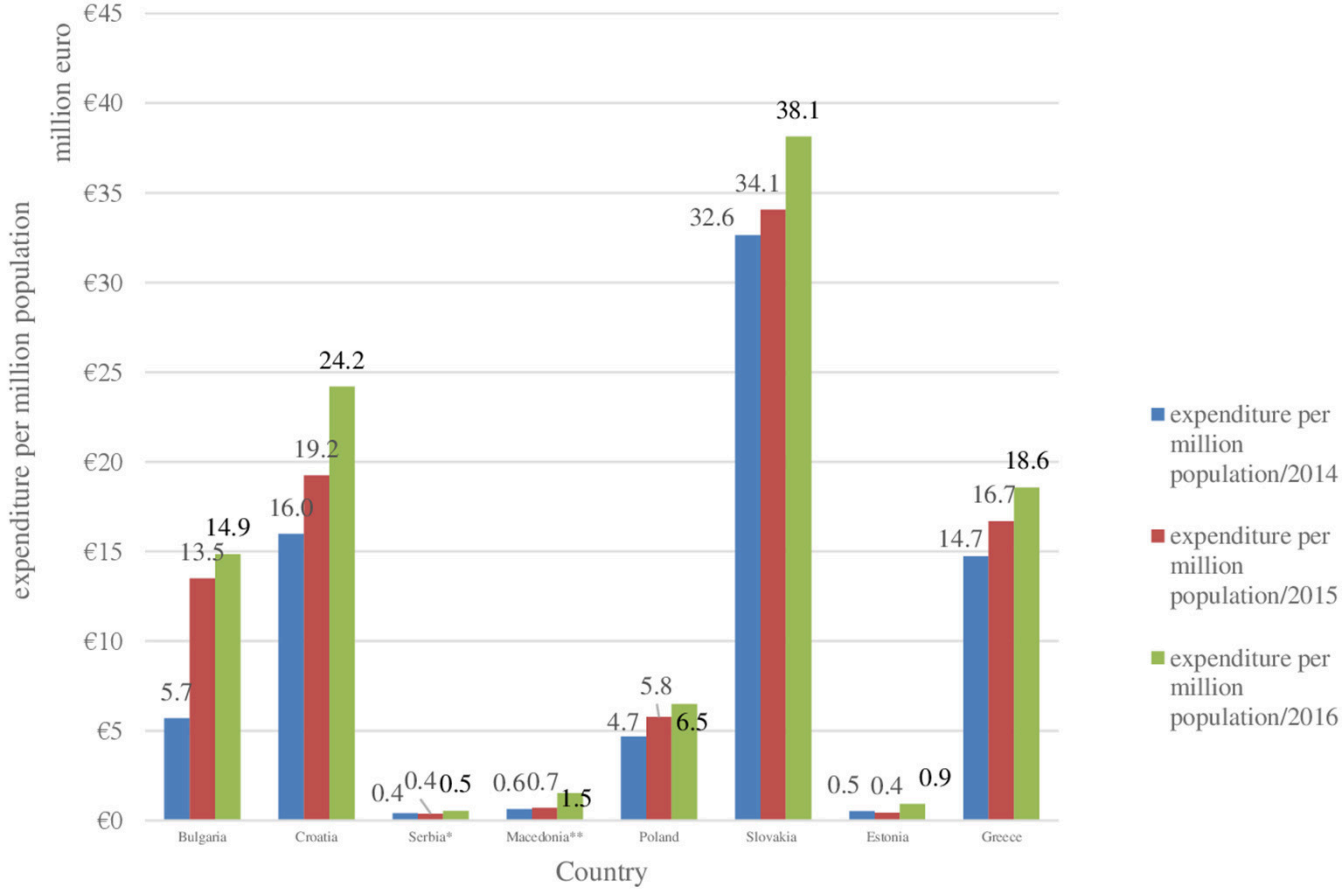
Total expenditures per million population paid by the public fund for biotechnological drugs for rare disease in each country in 2014–2016.

Total pharmaceutical expenditures covered by the local public fund have increased for 3-year period of time (2014–2016) in all countries, included in the survey (Table 5, Figures 2, 3). The same tendency is revealed for the pharmaceutical expenditures for biotechnological drugs for rare diseases and for their share of the total costs. Romania and Hungary were excluded due to lack of reliable data. Greece is characterized by relatively stable expenditures and it is the only country in which the total reimbursement expenditures decreased in 2015 in comparison with 2014. The lowest are both total reimbursed costs and the reimbursed costs for biotechnological drugs for RDs in Macedonia, Serbia, and Estonia, followed by Bulgaria, Croatia, and Slovakia. The highest are the costs in Poland and Greece in the observed period as their average values are ~214 million and 180 million euro, respectively. However, the expenditures per million populations are the highest in Slovakia (average value for the observed period−35million euro), followed by Croatia, Greece, and Bulgaria (Figure 3).

**Table 5:** Pharmaceutical expenditures in euro and share of biotechnological costs for rare diseases for 3 year period 2014–2016.

	**2014**			**2015**			**2016**		
**Country**	**Total pharmaceutical expenditures paid by the public fund(s)**	**Total costs for biotechnological drugs for rare diseases**	**The share of biotechnological drugs of total budget**	**Total pharmaceutical expenditures paid by the public fund(s)**	**Total costs for biotechnological drugs for rare diseases**	**The share of biotechnological drugs of the total budget**	**Total pharmaceutical expenditures paid by the public fund(s)**	**Total costs for biotechnological drugs for rare diseases**	**The share of biotechnological drugs of the total budget**
Bulgaria	544,014,561 €	4,105,545,097 €	8%	559,353,318 €	9,704,490,620 €	17%	560,000,000€	10,553,494,188 €	19%
Croatia	667,451,350 €	67,495,070 €	10.10%	702,258,400 €	80,611,350 €	11.50%	773,730,950 €	100,484,260 €	13.00%
Serbia^*^	553,222,657 €	2,858,524 €^*^	0.52%	521,659,251 €	2,729,507€^*^	0.52%	532,952,966 €	3,815,050 €^*^	0.72%
Macedonia^**^	94,264,520 €^*^	1,286,174 €^**^	1.36%	96,875,485 € ^*^	1,446,945 € ^**^	1.5%	103,313,835 €^*^	3,167,203 €^**^	3%
Hungary^***^	~1.16 bn €	n/a	n/a	~1.25 bn €	n/a	n/a	~1.34 bn €	n/a	n/a
Poland	23,912,983,801 €	1,781,186,676 €	7.4%	2 559 558 859,3 €	2,190,791,117 €	8.60%	2 6,956,623,330 €	2,461,720,386 €	9.10%
Slovakia *(NCZI, 2017)*^a^, ^b^	1,052,300,000 €	176,893,112 €	16.80%	1,111,100,000 €	184,782,533 €	16.60%	1,179,700,000 €	207,263,289 €	17.60%
Estonia	109,753,000 €	685 237 €	0.62%	112 801 000 €	587 507 €	0.52%	131, 246,000 €	1,201,548 €	0.92%
Greece	2.0 Bn €	160 mio €	8%	2.0 Bn €	180 mio €	9%	1.945 Bn €	200 mio €	10%
Romania	2,105,955,111 €			2,063, 033,777 €			2,114,979,555 €		

* *Budget line for “rare diseases medicines”, not all reimbursed biotechnological drugs for rare disease are in that budget line. This is only available data. Values in EUR calculated based on average exchange rate for 2014 (1 EUR = 117,3060 RSD), 2015 (1 EUR = 120,7328 RSD) and 2016 (1 EUR = 123,1179 RSD)*.

** *Source: annual report of the Health Insurance Fund; Data from work reports of Association of people with rare diseases and Programs of rare diseases by the Ministry of Health*.

*** *These expenditures are the total budgets on reimbursed pharmaceuticals. However, there are special medicines (e.g., orphan drugs, biologic and biosimilar medicines) whose prices are not publicly available due to tendering. Therefore, these are only approximate values on the total pharmaceutical expenditures*.

a *Total pharmaceutical expenditures paid by public fund(s) in Slovakia. Národné centrum zdravotníckych informácií, Bratislava 2017. http://www.nczisk.sk/Documents/publikacie/2016/sp1701.pdf (Accessed January 15, 2018)*.

b *Total costs for biotechnological drugs for rare diseases in Slovakia: http://www.mcr.sk/?lang=en, Spotreba liekov na Slovensku, Modra 2000-2017, MCR, s.r.o. 2017, MCR, s.r.o*.

**Figure 4:**
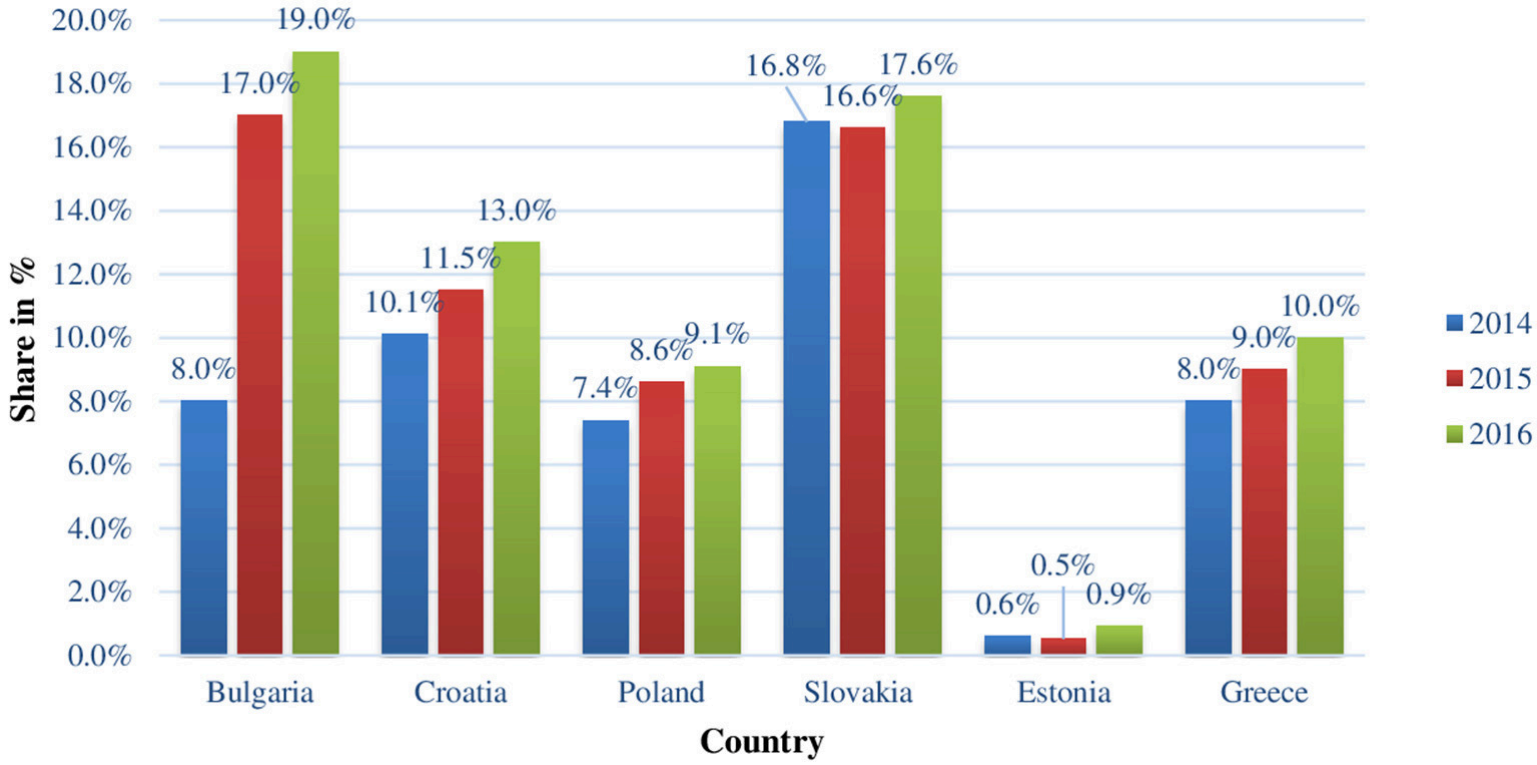
Share of the costs for biotechnological drugs for rare diseases of the total reimbursed pharmaceutical costs in all CEEC for the period 2014–2016.

The shares of expenditures on the reimbursement of biotechnological drugs for RDs in the individual countries vary between 0.5% in Serbia and 16.8% in Slovakia in 2014. Similar variation is observed in 2015 and 2016: between 0.52% in Serbia and Estonia and 16.6% in Slovakia and between 0.7% in Serbia and 19% in Bulgaria, respectively for both years. The most significant is the increasing share of biotechnological drugs in Bulgaria: from 8% in 2014 to 19% in 2016 (Figure 4).

## Discussion

To the best of our knowledge this is the first study comparing the legislative requirements, patients' access to biotechnological medicines for rare diseases and financial impact of these medicinal products on the pharmaceutical budget of low- and middle-income countries from Central and Eastern Europe (CEECs). Our work is the first to make such a comparison and produce significant results that can be used as a basis for conducting further detailed studies among more countries in the region, taking into account the level of reimbursement, co-payment, and dynamics of the number of BOMPs in local markets. Our study provides new evidence about the access to orphan medicines in countries with similar macroeconomics that was gathered over a period of 3 years and confirms previously published statements that orphan legislation principles as well as the availability and accessibility of ODs vary among the EU countries (Iskrov et al., 2012; Logviss et al., 2016). The study is based on expert opinion and evidence from the literature (Vogler et al., 2015; Kawalec et al., 2017) (Baltic guideline)^5^.

Despite the implementation of relevant legislation and pharmacoeconomic criteria for cost-effectiveness assessment of BOMPs, their number varied significantly among CEECs. This could influence the access to appropriate treatment and could worsen patients' condition and reduce significantly the quality of life of patients with rare diseases for whom innovative biotechnological medicines are not reimbursed. Limited access to adequate therapy could lead to additional healthcare costs as well as to reduced productivity and losses for the society as a whole. Most of the biotechnological medicines for RDs reimbursed in CEECs belong to the group of antineoplastic and immunomodulating agents according to ATC classification system, which demonstrates the increasing impact of oncological diseases on public healthcare spending. Our analysis shows that the access to BOMPs in all CEECs has been improving in the recent years and the countries with the best access are Greece and Hungary. What is more, we can acknowledge that the number of reimbursed orphan drugs in a given country is correlated with the time of joining the EU. Orphan drugs are reimbursed to a lesser extent in the new EU member states, which could be explained by their limited healthcare spending as compared with the countries that joined EU earlier (de Varax et al., 2004; Pavlović et al., 2013). Our results are in accordance with the results of another study in which it is shown that Greece ensures more adequate access to orphan drugs than Romania and Bulgaria (Kamusheva et al., 2013). Stoimenova et al. analyzed the access to orphan medicines in Bulgaria and the share of biotechnology-derived products reimbursed for rare diseases in Bulgaria in 2011 (Stoimenova et al., 2011) Comparing our results with the results of this study, it is obvious that the access to biotechnology-derived medicines which are intended to treat rare diseases has been improving in Bulgaria in the recent years from around 13 in 2011 to 36 in 2017. The number of reimbursed BOMPs in Serbia is limited probably due to incomplete compliance with European Union legislation (Pavlović et al., 2013) and high prices of BOMPs, which is considered a limiting factor for their inclusion in the positive drug list (Pejčić and Iskrov, 2016).

Additional approaches for ensuring access to BOMPs through other mechanisms are reported only in Hungary. An example of such mechanism is the patient-based reimbursement system. Hungarian patients have the possibility to receive reimbursement for high-priced medicines not included in the reimbursement list through a special budget allocated on an individual basis (Németh et al., 2017). Patients with rare diseases in all other countries included in the study are provided appropriate treatment through hospital budgets and local health insurance funds. Macedonia, as a non-EU member state, faces a lot of challenges in ensuring access and affordability to BOMPs, which is probably associated with the lack of adequate financing (Zlatareva et al., 2013).

The financial burden of BOMPs is significant in CEECs considering limited funding for pharmaceuticals in these countries, especially non-European member states, Macedonia and Serbia. The main cost driver is the price of the biotechnological medicines resulting from the innovative methods used for their manufacturing. Reimbursement of these high-priced groups of medicines is also determined by their ability to fulfill the unmet medical needs, especially of patients with rare diseases. The inclusion of the so-called biosimilars, which are as safe and effective as the reference medicine (Dowlat et al., 2016), might significantly reduce the financial burden of biotechnological medicines. As Dowlat state orphan medicines form a significant part of the healthcare spending because of their high cost, which is also the main reason for a drug not always to be reimbursed (Dowlat, 2016). Therefore, the opportunity for the development of biosimilars for BOMPs should be highlighted. Moreover, Dowlat et al. emphasizes that biosimilar development would be gradual due to legislative hurdles, market and data protection and exclusivity of the originators and the protection of intellectual property.

All these inequalities between the CEECs regarding BOMPs and biotechnological MPs for RDs could be explained with the differences in the reimbursement approaches, but mostly due to the financial restrictions determined by the governments. Shared projects and collaboration among the CEECs could improve the national reimbursement systems and enhance the knowledge of national experts for the purposes of preparing more precise and valuable pharmacoeconomic analyses for biotechnological orphan medicines. The adaption of well-working risk-sharing schemes, patients oriented legislative changes in the healthcare sector, exploring and implementation of the best reimbursement practices available worldwide could significantly improve the patients' access to biotechnological treatment in poorer performing CEECs.

## Conclusion

Our comparative analysis showed that despite the limited number of patients with rare diseases, biotechnological medicines for such diseases have a significant impact on pharmaceutical spending of each CEEC. The tendency is for slow increase in the share of BOMPs and BMPs for RDs of the total costs (in some countries, e.g., Bulgaria, the increase was two-fold). Therefore, the access to these medicines and their affordability to the patients have been improving in the recent years, which is especially visible in Hungary and Greece. However, the non-European Union CEECs such as Macedonia face a delay in the legal implementation of a pharmacoeconomic guidelines for the assessment of BOMPs. Despite the fact that Serbia is not in the EU, its legislation in the area of reimbursement of BOMPs and the level of patients' access to treatment is similar to that of Bulgaria, Slovakia and Estonia. The CEECs have similar reimbursement requirements and there is a tendency for developing the systems in line with the latest scientific, pharmacoeconomic, and health technology assessment guidelines.

## Author contributions

GP defined the goal of the study, its conception and design. MK prepared the questionnaires and coordinated the project. MK, AS, MM, KM, and GP carried out the interpretation of data and prepared the draft of the manuscript. AH, ZK, MK, PK, BA, DL, TT, PD, MG, MH, MP, and AM collected input data for all corresponding CEE countries. All contributed to editing and approving the final version of the manuscript.

### Conflict of interest statement

The authors declare that the research was conducted in the absence of any commercial or financial relationships that could be construed as a potential conflict of interest.

## Footnotes

^1^WHO report, Priority diseases and reasons for inclusion. 6.19

^2^Rare diseases. Available online at: http://www.who.int/medicines/areas/priority_medicines/Ch6_19Rare.pdf; http://static.correofarmaceutico.com/docs/2016/12/12/qiihi_outlook_for_global_medicines_through_2021.pdf

^3^Outlook for Global Medicines through 2021. Balancing Cost and Value. QuintilesIMS Institute. 2016.

^4^Orphanet List of Orphan drugs, July 2017.Avaialble online at: www.orphanet.net

^5^Baltic guideline for pharmacoeconomic assessment, https://www.sm.ee/sites/default/files/content-editors/eesmargid_ja_tegevused/Tervis/Ravimid/balti_juhis_ravimite_farmakooekonoomiliseks_hindamiseks.pdf

## Abbreviations

ATC: Anatomical Therapeutic Chemical Classification
BOMPs: Biotechnological Orphan Medicinal Products
CEECs: Central and Eastern European Countries
EU: European Union
GDP: Gross Domestic Product
HTA: Health Technology Assessment
HAS: Haute Autorité de Santé
INN: International Nonproprietary Name
IQWIG: The Institute for Quality and Efficiency in Healthcare
NICE: National Institute for Health and Care Excellence
RDs: Rare Diseases.
